# Experimental icing affects growth, mortality, and flowering in a high Arctic dwarf shrub

**DOI:** 10.1002/ece3.2023

**Published:** 2016-02-28

**Authors:** Jos M. Milner, Øystein Varpe, René van der Wal, Brage Bremset Hansen

**Affiliations:** ^1^ School of Biological SciencesUniversity of Aberdeen23 St. Machar DriveAberdeenAB24 3UUU.K; ^2^ University Centre in Svalbard9171LongyearbyenNorway; ^3^ Akvaplan‐nivaFram Centre9296TromsøNorway; ^4^ Centre for Biodiversity DynamicsDepartment of BiologyNorwegian University of Science and Technology7491TrondheimNorway

**Keywords:** Anoxia tolerance, *Cassiope tetragona*, climate change, extreme weather event, resource allocation, winter warming

## Abstract

Effects of climate change are predicted to be greatest at high latitudes, with more pronounced warming in winter than summer. Extreme mid‐winter warm spells and heavy rain‐on‐snow events are already increasing in frequency in the Arctic, with implications for snow‐pack and ground‐ice formation. These may in turn affect key components of Arctic ecosystems. However, the fitness consequences of extreme winter weather events for tundra plants are not well understood, especially in the high Arctic. We simulated an extreme mid‐winter rain‐on‐snow event at a field site in high Arctic Svalbard (78°N) by experimentally encasing tundra vegetation in ice. After the subsequent growing season, we measured the effects of icing on growth and fitness indices in the common tundra plant, Arctic bell‐heather (*Cassiope tetragona*). The suitability of this species for retrospective growth analysis enabled us to compare shoot growth in pre and postmanipulation years in icing treatment and control plants, as well as shoot survival and flowering. Plants from icing treatment plots had higher shoot mortality and lower flowering success than controls. At the individual sample level, heavily flowering plants invested less in shoot growth than nonflowering plants, while shoot growth was positively related to the degree of shoot mortality. Therefore, contrary to expectation, undamaged shoots showed enhanced growth in ice treatment plants. This suggests that following damage, aboveground resources were allocated to the few remaining undamaged meristems. The enhanced shoot growth measured in our icing treatment plants has implications for climate studies based on retrospective analyses of *Cassiope*. As shoot growth in this species responds positively to summer warming, it also highlights a potentially complex interaction between summer and winter conditions. By documenting strong effects of icing on growth and reproduction of a widespread tundra plant, our study contributes to an understanding of Arctic plant responses to projected changes in winter climatic conditions.

## Introduction

Effects of climate change are predicted to be greatest at high latitudes, with Arctic warming being more pronounced in winter than summer (IPCC, 2014) and extreme events becoming more frequent (Jentsch et al. 2007; Rennert et al. 2009). In addition to a long‐term warming trend, changes to winter weather patterns include the occurrence of short but warm spells in mid‐winter (Bokhorst et al. 2010, 2011) and heavy ROS (rain‐on‐snow) events (Putkonen and Roe 2003), both of which are already increasing in frequency in some regions (Bokhorst et al. 2008; Rennert et al. 2009; Hansen et al. 2014). Such extreme weather events have implications for snow‐pack and permafrost conditions and may lead to the formation of ground‐ice (Putkonen and Roe 2003; Westermann et al. 2011). An increasing body of evidence suggests that ground‐ice resulting from extreme winter events may in turn affect a multitude of Arctic ecosystem components (Coulson et al. 2000; Hansen et al. 2013; Cooper 2014; Convey et al. 2015) and is one of the major environmental changes affecting Arctic terrestrial ecosystems (Ims and Ehrich 2013).

Winter precipitation is projected to increase across much of the Arctic (Bintanja and Selten 2014). If the additional precipitation falls as snow, it will lead to deeper and prolonged snow‐packs (Mallik et al. 2011; Blok et al. 2015). Such conditions provide good insulation for plants and soils during winter but reduce the length of the growing season, give colder, wetter soils, delay plant phenology and decrease reproductive success of vascular plants (Wipf and Rixen 2010; Cooper et al. 2011). However, if precipitation coincides with spells of mild winter weather, it will fall as rain, leading to snow melt and reduced snow‐pack depth (Bokhorst et al. 2009). Although this scenario suggests a longer growing season due to earlier snow melt (Van Wijk et al. 2003), it also compromises the protective insulation cover that snow provides to underlying plants and may break their physiological winter hardening (Bokhorst et al. 2010; Semenchuk et al. 2013). Consequently, plants become vulnerable to damage when winter conditions return to normal or during late spring frosts (Bokhorst et al. 2009, 2011). These negative effects tend to outweigh the benefits of a longer growing season (Wipf et al. 2009). As well as reducing snow‐pack depth, mid‐winter rain increases subsnowpack soil temperatures and causes ground‐ice to form (Putkonen and Roe 2003; Hansen et al. 2014). Ground‐ice and ice layers in the snow can reduce herbivores' access to vegetation beneath the ice layer, triggering large‐scale mortality and disrupting the population dynamics of terrestrial animals (Forchhammer and Boertmann 1993; Kohler and Aanes 2004; Hansen et al. 2011, 2013). Besides reducing access to forage plants, little is known about the consequences of rain‐on‐snow or ice encasement for the plants themselves (Preece et al. 2012; Preece and Phoenix 2014). This is partly because climate change studies of Arctic vascular plants have, until recently, focused on responses to changes in summer rather than winter conditions (Bokhorst et al. 2011; Cooper 2014).

Ice encasement imposes low oxygen conditions on plants (Schluter and Crawford 2003) and can cause damage as a result of cellular dehydration and acidosis (Preece and Phoenix 2014). If ice‐encased plants switch from aerobic to anaerobic respiration, cell death can occur when toxic levels of by‐products, including carbon dioxide, accumulate (Gudleifsson & Bjarnadottir, 2014). In addition, cell membranes may be damaged during rapid oxidation as tissues are reexposed to air when the ice melts (Crawford et al. 1994). However, studies of the effects of ice encasement on dwarf shrubs (*Vaccinium uliginosum*,* V. vitis‐idaea*, and *Empetrum nigrum*) in the sub‐Arctic have shown that these species are relatively tolerant of icing (Preece and Phoenix 2013, 2014). Low metabolic activity during winter is likely to play a role in this (Preece et al. 2012; Preece and Phoenix 2014), although earlier studies suggested that arctic plants may also have a high tolerance of anoxia (Crawford et al. 1994; Crawford 2014). To our knowledge, no field studies have examined the effects of icing on tundra plants in the high Arctic, despite the vulnerability of this ecosystem to icing events (Hansen et al. 2013).

In this field study, we simulated an extreme mid‐winter rain‐on‐snow event by experimental ice encasement of tundra vegetation dominated by Arctic bell‐heather *Cassiope tetragona* (hereafter *Cassiope*). This widespread and common dwarf shrub is particularly suited to studies of climate effects as its annual growth and reproduction can be analyzed retrospectively (Callaghan et al. 1989; Johnstone and Henry 1997; Weijers et al. 2012). While a number of studies have documented a positive response in leaf and shoot growth of *Cassiope* to increasing summer temperatures (Callaghan et al. 1989; Havström et al. 1993; Rozema et al. 2009; Weijers et al. 2010), the extent to which winter climate change influences growth and reproduction is far from understood. Given that winter warming may induce particularly severe changes in high Arctic environments in the future, its ecological effects are understudied and potentially underestimated (Post et al. 2009). The aim of our experiment was therefore to investigate the effects of ice encasement on indices of *Cassiope* fitness during the subsequent growing season, using control and icing treatment plots while accounting for individual‐level past growth and reproduction. Based on previous experimental manipulations in the sub‐Arctic (Bokhorst et al. 2008, 2010, 2011; Preece and Phoenix 2013, 2014), we expected icing treatment plants to show higher shoot mortality (due to damage to vegetative buds) and lower flowering success (due to damage to reproductive buds during winter). We also expected reduced annual shoot growth in the icing treatment compared with controls as a result of hypoxia damage. If evident, changes in growth and reproduction patterns would have implications for the use of *Cassiope* as a bioindicator of climate change, as well as overall relevance for our predictive understanding of the ecological effects of increasingly warm and wet winters in the Arctic.

## Methods

### Study species

*Cassiope* is a long‐lived ericaceous evergreen dwarf shrub. It is a dominant species of the Arctic tundra, with a circumpolar distribution. It occurs on dry heaths and fellfields, particularly in sheltered snow beds with moderate to high snow accumulation (Callaghan et al. 1989; Johnstone and Henry 1997). Snow cover protects *Cassiope* plants from extreme temperatures suggesting it may be less cold‐tolerant than other dwarf shrubs such as mountain avens, *Dryas octopetala,* which occurs on snow‐blown ridges exposed to low temperatures. *Cassiope* grows monopodially with initially upright, then creeping, shoots (Johnstone and Henry 1997). Branches are usually produced near the base of an annual growth increment, just below any flowers which may be present (Havström et al. 1993). However, there are high levels of individual variability believed to result from within‐plant resource partitioning, plant architecture, and associated micro‐environmental conditions (Rayback & Henry, 2005).

### Study area

**Figure 1:**
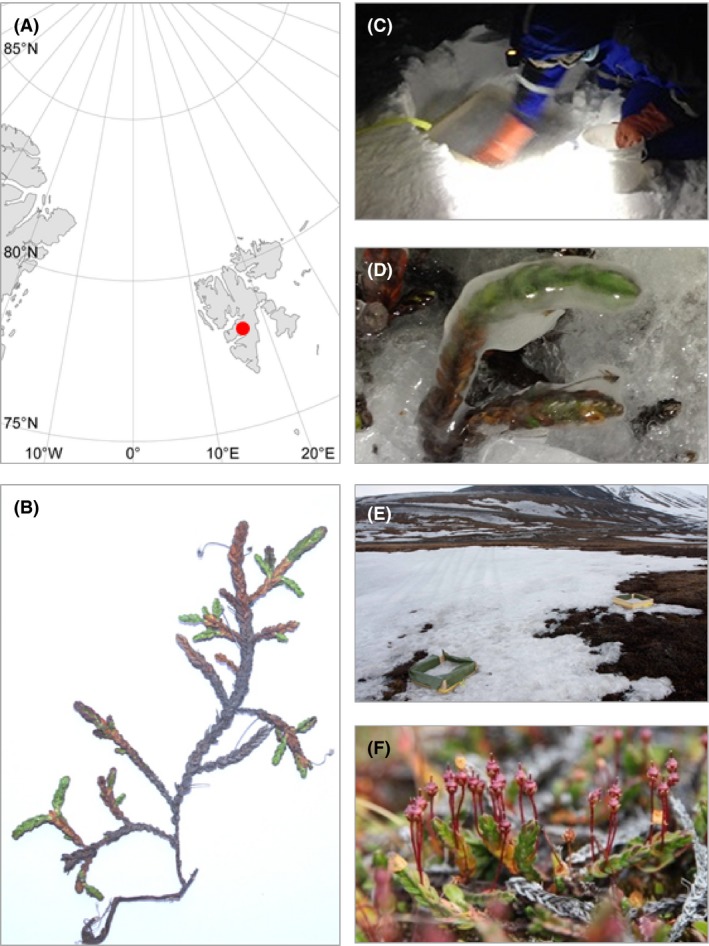
(A) Map showing location of study area in Adventdalen, Svalbard (red spot) with Greenland to the west. (B) Sampled *Cassiope tetragona* ramet showing dead (brown) and live (green) shoots, including new lateral green shoots from the 2014 growing season and flowers from earlier years (gray flowers). (C) Creating an icing treatment plot during “polar night” in January 2014. (D) *C. tetragona* shoots in treatment plot during the process of ice encasement. (E) Treatment plot during spring snow melt in June 2014. (F) *C. tetragona* flower buds in control plot in July 2014.

Our study area was at the mouth of Bolterdalen (78°16′N 15°99′E), a side valley of Adventdalen, near Longyearbyen, Svalbard, at an altitude of approximately 100 m above sea level (Fig. 1A). It has a maritime Arctic climate. At Svalbard Airport, 14 km away, the 30‐year (1961–90) mean winter (November–April) total precipitation and temperature were 113 mm and −12.7°C, respectively. However, warm spells and winter rain occur relatively frequently despite the high latitude and represent an increasingly common weather phenomenon (Hansen et al. 2014). The study area was located on a gentle west‐facing slope (3–4°) covered with *Cassiope* heath (Fig. 1B and E) characterized by *Cassiope* and bryophytes with some polar willow, *Salix polaris*, the woodrush *Luzula confusa* and *D*. *octopetala*. Being located close to a windblown ridge, the degree of snow accumulation in our study area was relatively low for *Cassiope* heath (e.g., Blok et al. 2015), with snow depths of approximately 20–40 cm at the time of the experimental manipulation.

### Experimental design and sampling

The experiment was set up on 8–9th January 2014, during the polar night. The study area was chosen partly based on its proximity to the road because of the logistical constraints of simulating heavy rain in the field during the dark and cold Arctic winter. Based on terrain characteristics, six pairs of plots were laid out in a focal area along a 60 m band of *Cassiope* heath vegetation. Each plot pair was at least 5 m apart to limit the possibility of sampling the same individual across plots and had a high abundance of *Cassiope* under the snow‐pack, confirmed using a shovel. Within each pair, one plot was chosen for experimental icing in fairly continuous *Cassiope* vegetation and on relatively even ground to enable ice encasement. The other plot, with similar vegetation structure and micro‐topography, was allocated as a control, 3–5 m away along the same contour. After removing the snow, a 50 cm × 50 cm wooden frame, 13 cm high, was placed on the ground in each icing plot (Fig. 1C). Approximately 40 l of cold (0–2°C) water was brought to each icing plot by sledge. The water was cooled to almost 0°C by mixing it with snow in a bucket, and carefully poured into the frame such that all plant parts became encapsulated in a first thin layer of solid ice (Fig. 1D). The frame was then gradually filled with a mixture of snow slush and cold water over a two‐day period when average temperatures at Svalbard airport were −15 and −7°C, respectively. By the end of the second day, the treatment had successfully created a 10 cm partly solid and partly porous ice layer, covering all *Cassiope* plant parts within the frame. Based on visual appearance, this mimicked the natural ice encasement we have observed in the region in some years (Hansen et al. 2014). Treatment plots were topped up with snow as necessary to achieve a similar snow depth across all experimental plots.

The plots were revisited to remove the wooden frames during snow melt (22nd June 2014; Fig. 1E). *Cassiope* plants from both treatment and control plots were sampled on 17–18th September 2014 to evaluate the impact of the icing treatment after one growing season. Within plots, we sampled plants using a 50 cm × 50 cm sampling quadrat which was subdivided into 25 smaller squares. We sampled the closest live (with at least one green shoot) ramet to each of five pre‐defined string intersections, leaving a 10 cm unsampled buffer around the edge of the plot to avoid potential edge effects associated with the icing treatment. Thus, we collected 60 samples for subsequent analysis (5 per plot × 2 treatments (control and icing) × 6 pairs). All sampled plants were air dried at room temperature for 1 week and then kept frozen until analysis.

### Shoot mortality and flowering

Shoot mortality was quantified by counting the total number of live (green) and dead (brown or gray, lacking any green leaves) shoots on sampled ramets (Fig. 1B). The total number of shoots per sample ranged from 14 to 119 (mean ± SE = 53 ± 3.4). A number of dominant shoots showed clusters of new green lateral shoots around an apparently dead or damaged apical shoot tip (Fig. 1B). These and the number of new lateral shoots were also counted. We quantified flower production, a proxy for fecundity (sensu Johnstone and Henry 1997), by counting the number of flowers per sample, broken down into those of the current (2014) growing season (with a reddish peduncle and flower generally intact with whitish petals) and older flowers (occurring further down the shoot, with a gray peduncle and gray or broken off flowers; Fig. 1B). The total number of flowers per sample ranged from 0 to 139 (mean ± SE = 26 ± 3.9).

### Shoot growth

*Cassiope* is well known for its suitability for retrospective growth analysis based on wave‐like patterns of seasonal growth in leaf length and distances between adjacent leaf nodes (Callaghan et al. 1989; Weijers et al. 2012). The shortest internode length of each wave corresponds with the end of each growing season (Johnstone and Henry 1997), allowing the annual growth increment of a shoot to be measured as the distance between consecutive internodal minima. To investigate the effect of treatment on apical shoot growth, we used this approach to measure the annual increments in the manipulation year (2014) and three previous years (2011–2013) for two shoots from each of three randomly chosen samples from each treatment at each plot pair. Therefore, in total, we measured four growth increments on each of 72 shoots (2 shoots × 3 samples (ramets) per plot × 2 treatments (control and icing) × 6 pairs). Only undamaged, dominant apical shoots which could be dated back at least 4 years were used. Shoots were examined under a dissecting microscope at ×10 magnification and increments were measured to the nearest 0.1 mm.

### Statistical analysis

We analyzed the plants' responses to the experimental treatment in terms of numbers of dead shoots and the current season's flowers, using GEE (generalized estimating equation) models (Diggle et al. 2002). GEE models accounted for nonindependence within plots and the heterogeneity among observations which gave rise to overdispersion (variance > mean) in our counts (Zuur et al. 2009) and heteroscedastic model residuals in preliminary analyses carried out using Poisson generalized linear mixed regression (not presented). Counts were modeled within R, version 3.1.2 (R Core Team, 2014), using the geeglm function from the geepack package. We specified a Poisson distribution and fitted treatment (icing or control) as a categorical explanatory variable. To account for spatial dependency in the data, plot ID was used as the grouping structure, and we specified the error correlation structure as ‘exchangeable' (Zuur et al. 2009). The total number of shoots or flowers on each sample was fitted as an offset to account for between‐sample differences in the amount of material collected.

AGI (Annual growth increments) in each year were expressed relative to the premanipulation mean AGI for that shoot (i.e., in year *t*, relative annual growth increment [rAGI_*t*_] = AGI_*t*_/mean[AGI_2011–2013_]) to account for variation in individual shoot length. To determine shoot growth response to the treatment, we compared our index of relative growth in each of the 4 years measured (rAGI_*t*_) between shoots from icing and control plots using LME (linear mixed‐effects) modeling. Our expectation was that relative growth would be similar between treatments in the first 3 years (premanipulation) and differ between treatments in the last year (postmanipulation), giving rise to a year─treatment interaction. Furthermore, we expected lower postmanipulation growth in icing treatment plants than control plants. We used the lme function in the nlme package in R and fitted the growth year (4‐level factor), treatment (2‐level factor), and their interaction as fixed effects. We fitted plot ID as a random effect. Focusing specifically on postmanipulation growth, we also related rAGI_2014_ of an individual ramet (averaged across the two shoots) to the proportion of its shoots that were dead and the proportion of its flowers that were from 2014. The proportions of dead shoots and 2014 flowers, and their interactions with treatment, were fitted as fixed effects and plot ID was fitted as a random effect in a LME model.

## Results

### Shoot damage and mortality

**Figure 2:**
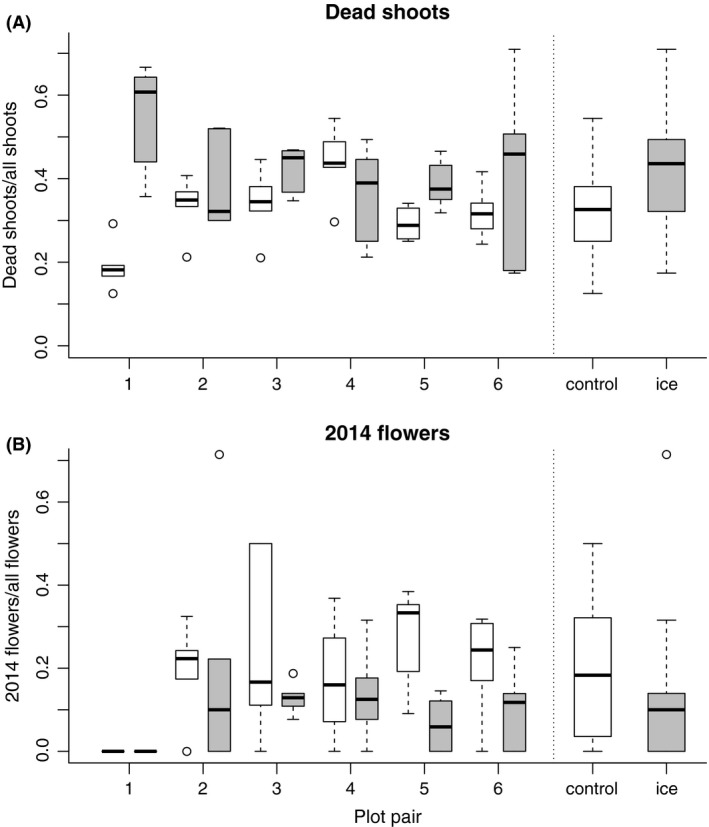
The effects of icing on *Cassiope tetragona* plants at each of six experimental plot pairs in Adventdalen, Svalbard (left) and averaged across all plots (right). Comparisons are of paired icing (gray) and control (white) plots showing (A) the proportion of all recorded shoots that were dead and (B) the proportion of all recorded flowers that were from the 2014 growing season (for classification into 2014 vs. older flowers, see Methods), both measured at the individual plant sample level. Boxes show the first to third quartile range with median (thick horizontal line). Dashed lines give the minimum and maximum values except where there are outliers (open dots), in which case they show 1.5 times the interquartile range.

Plants from ice treatment plots had a high proportion of dead shoots (mean ± SE: 0.42 ± 0.03) compared with those from controls (mean ± SE: 0.32 ± 0.02; Fig. 2A). The difference between treatments was significant after accounting for the number of shoots present (Wald statistic = 4.36, *P *=* *0.037). There were 10 samples in which some shoots appeared to be dying (showing a mixture of dead and greenish‐yellow leaves) or were dead but carried flowers from the 2014 growing season. All these samples came from icing treatment plots suggesting that the viability of surviving shoots may also have been compromised by the icing treatment.

Dead or damaged shoot tips with a cluster of multiple new lateral shoots were a visually striking feature of plants sampled in this experiment (Fig. 1B). The number of damaged shoots was negatively related to the number of dead shoots (Wald statistic = 5.86, *P *=* *0.016) suggesting that in some plots, less vulnerable shoots were damaged rather than killed. The proportion of samples with at least one damaged shoot did not differ significantly between treatments (0.63 of control and 0.77 of icing samples) but the combined number of dead and damaged shoots was significantly greater in the icing treatment (0.36 and 0.45 of all shoots in control and icing samples, respectively; Wald statistic = 5.29, *P *=* *0.022).

### Flowering

There was a clear difference in flower production between treatments, with icing treatment plants producing fewer flowers in 2014, the summer following manipulation, than controls: 90 flowers summed across all icing plots compared with 174 flowers in all control plots (Fig. 2B). However, plot pair one differed from the other five plot pairs in having no 2014 flowers on any of the sampled plants in either treatment. This pair also had very few older flowers from previous (premanipulation) years: a total of three and nine old flowers in control and icing treatment samples, respectively at plot pair one, compared with 67‐224 (control) and 63‐210 (icing) old flowers at the other five pairs. Omitting the aberrant plot pair one, there was a highly significant negative effect of the icing treatment on flowering in 2014 (Wald statistic = 35.6, *P *<* *0.001).

### Shoot growth

**Figure 3:**
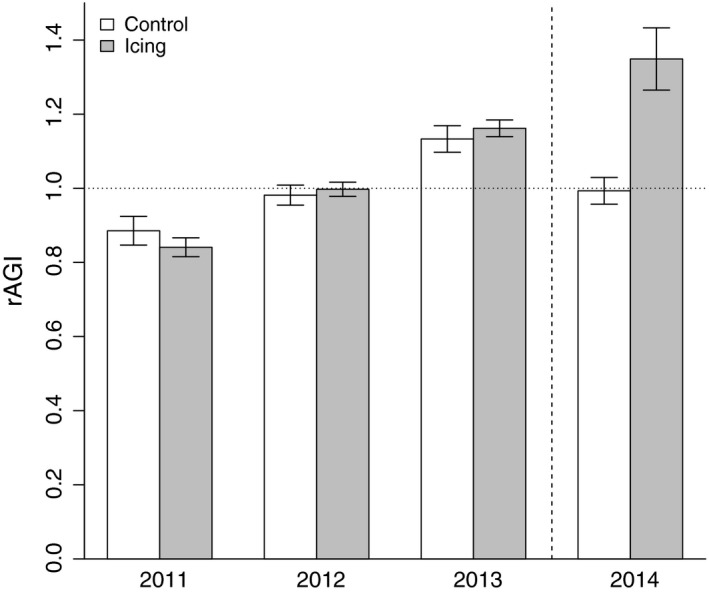
Mean (±1 SE) relative annual growth increments (rAGI) of *Cassiope tetragona* shoots in 3 years premanipulation (2011–2013) and in the postmanipulation year (2014) in plots assigned to experimental icing (gray) or control (white) treatment in 2014 (represented by vertical dashed line). The horizontal dotted line represents the average growth in premanipulation years.

**Figure 4:**
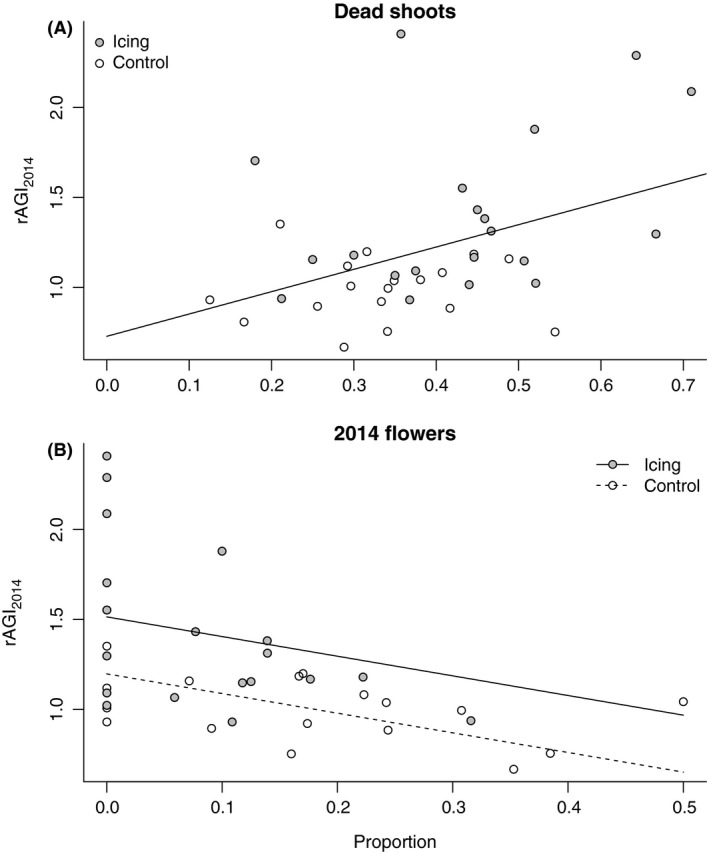
Relative annual growth increments of *Cassiope tetragona* in 2014 (rAGI_2014_) in relation to (A) the proportion of all recorded shoots that were dead and (B) the proportion of all recorded flowers that were from the 2014 growing season (for classification into 2014 vs. older flowers, see Methods), both measured at the individual plant sample level.

The relative growth of *Cassiope* shoots (rAGI) varied significantly between years (Likelihood ratio = 62.4, *P *<* *0.001) but during the premanipulation period, 2011–2013, there was consistency in the growth of shoots from all plots in any given year, regardless of subsequent treatment (e.g., below average growth in 2011 and above average in 2013 in both icing and control plots; Fig. 3). Consequently, during the premanipulation period, there was no significant interaction between year and sub‐sequent treatment (LR = 1.88, *P *=* *0.39) or significant difference in growth between plots in relation to subsequent treatment (LR < 0.001, *P *≈* *1). However, following the manipulation experiment in 2014, there was high relative growth in the plants from the icing treatment (1.35 ± 0.08) compared with average growth in the control plants (0.99 ± 0.04). This led to a significant treatment─year interaction across the 4 years (Likelihood ratio = 28.7, *P *<* *0.001; Fig. 3). Furthermore, within individual plant samples, shoot growth following manipulation, rAGI_2014_, was positively correlated with the proportion of dead shoots (*r *=* *0.415, *P *=* *0.010) and negatively correlated with the proportion of flowers that were produced in 2014 (*r *=* *−0.50, *P *=* *0.002; Fig. 4). Treatment and the proportion of flowers from 2014 explained significant variation in shoot growth within a sample (Likelihood ratio = 5.30, *P *=* *0.021; Likelihood ratio = 4.99, *P *=* *0.026 respectively), but the proportion of dead shoots explained no additional variation after accounting for treatment and flowering. There was also a tendency for reduced flowering to be more strongly related to growth in icing than control plants (Likelihood ratio = 3.64, *P *=* *0.057). Collectively, these results showed that shoot growth was greater in individuals suffering high shoot mortality, typical of the icing treatment, and lower in individuals that flowered more heavily, particularly among controls.

## Discussion

The predicted increase in frequency and magnitude of icing events represents one of the major environmental changes affecting Arctic terrestrial ecosystems (Ims and Ehrich 2013). This first study of icing effects on high Arctic vegetation showed clear consequences for both flowering success and shoot survival and growth of *Cassiope tetragona*. As expected, shoot survival (Fig. 2A) and flowering (Fig. 2B) were negatively affected by icing. However, shoot growth was positively related to the degree of shoot mortality (Fig. 4A), and heavily flowering plants invested less in shoot growth than nonflowering individuals (Fig. 4B). Accordingly, and contrary to our expectation, undamaged shoots showed enhanced growth in ice treatment plants (Fig. 3).

As *Cassiope* flower buds are likely set in the previous growing season (Semenchuk et al. 2013), the reduction in flowering among ice‐encased plants probably resulted from winter damage to existing buds rather than production of new ones. Our results are in line with observed reductions in *Cassiope* flowering following winters with natural warm spells (Semenchuk et al. 2013) and experimental winter icing in the sub‐Arctic (Preece et al. 2012; Preece and Phoenix 2014). Pan‐Arctic studies of (dwarf) shrub reproduction suggest a temperature dependency to recruitment pulses (Büntgen et al. 2015). However, recruitment peaked in the mid‐20th century and has been declining since despite rising temperatures (Büntgen et al. 2015). Our study, together with snow‐depth manipulation studies (Cooper et al. 2011; Mallik et al. 2011; Semenchuk et al. 2013), suggest that changes in winter climate adversely affect flowering success and could contribute to the observed decline in recruitment, despite warmer summers.

The higher shoot mortality and damage among icing treatment plants than controls support the suggestion that *Cassiope* may be less cold‐tolerant than species such as *D. octopetala*, adapted to exposed snow‐blown ridges (Semenchuk et al. 2013). As an evergreen shrub of snow beds, with overwintering flower buds near the tips of its erect shoots, *Cassiope* could be more vulnerable to freezing than other dwarf shrubs adapted to greater exposure, including lower‐lying, chamaephyte or deciduous species or those without overwintering flower buds (Preece and Phoenix 2013, 2014; Semenchuk et al. 2013). However, as our study area was at the northern limit of *Cassiope* distribution and has a relatively maritime climate, with frequent freeze‐thaw cycles, it is not clear how general our results are. Further studies are required to understand how *Cassiope* responds to rain‐on‐snow and icing in relation to latitude, topography, and microclimatic conditions. Furthermore, future studies should be conducted over several years to pick up any delayed effects and consequences for interannual variation in stem growth.

Leaf damage and defoliation can influence plant growth by remobilizing carbon and nitrogen reserves, leading to improved internal source‐sink relationships (Iqbal et al. 2012). Given that damage in our study occurred over winter, and that spring growth of Arctic plants is reliant on stored nutrients and carbon (Chapin et al. 2011), the unexpected enhanced shoot growth in treated plants may have arisen from reduced competition for relocated resources among few undamaged shoots. Observed trade‐offs between reproduction and growth have generally been attributed to resource limitation (Obeso, 2002), a condition typically experienced by high Arctic plants (Van der Wal and Hessen 2009). Indeed, *Cassiope* flower production has previously been reported to be negatively correlated with shoot growth in the same year (Johnstone and Henry 1997). However, whether the reduced flowering in our study contributed to the icing‐induced enhanced shoot growth is unknown, as there may not be a single resource limiting both reproduction and growth (Bonser and Aarssen 1996).

Icing‐induced enhancement of shoot growth has important implications for climate studies based on retrospective analyses of growth increments. Our result appears at odds with empirical observations of a tendency for low *Cassiope* growth following warm and wet winters in retrospective studies (Aanes et al. 2002; Weijers et al. 2012; Blok et al. 2015). However, this discrepancy may reflect the fact that *Cassiope* samples for retrospective analyses are generally well‐developed specimens, collected from deeper snow habitats where ground icing is uncommon, so as to maximize chronology length. Furthermore, enhancement of shoot growth depended on shoot mortality, but under natural snow bed conditions, shoot mortality would likely be lower than under our extreme experimental conditions so enhanced growth may not be detectable. Nonetheless, future studies of climate‐related changes in the growth of *Cassiope* and other Arctic shrub species should consider effects of both flowering and shoot mortality to avoid biased conclusions. Thus, while there is strong evidence of a positive effect of summer temperatures on *Cassiope* growth (Callaghan et al. 1989; Havström et al. 1993; Rozema et al. 2009; Weijers et al. 2010), potentially complex interactions between summer and winter conditions and the influence of icing on melt‐out and plant phenology are still far from understood. This may contribute to the absence of reported correlations between winter conditions and shrub growth (Blok et al. 2015), prior to our study.

The timing of extreme weather events may influence the severity of plant damage. Events in early to mid‐winter, as simulated here, prolong the period of ice encasement, potentially increasing the severity of effects of accumulated toxins (Preece and Phoenix 2014). On the other hand, winter frost hardening and dormancy dramatically affect plants' tolerance limits (Jentsch et al. 2007). Consequently, greater damage may occur if extreme warming occurs in late winter or spring when warmer temperatures and daylight coincide to break dormancy (Bokhorst et al. 2010, 2011). If soil thaw, associated with snowmelt (Van Wijk et al. 2003), has not occurred when plants start transpiring, transport from the roots is inhibited and water lost due to leaf activity cannot be replaced, causing desiccation and damage to apical meristems (Bokhorst et al. 2008, 2010). Our adverse icing effects could therefore have arisen from the long period of ice encasement, from damage due to early spring leaf activity while the roots were frozen, or a combination of both.

By documenting strong effects of experimental ice encasement on growth and an index of reproduction, our field study of *Cassiope* contributes to an understanding of the responses of Arctic plants to projected changes in climatic conditions. Previous work has tended to focus on the effects of warmer summers, but the consequences of changing conditions during the long Arctic winter are clearly important in understanding how global warming influences vegetation and ecosystem processes (Post et al. 2009; Cooper 2014). In particular, the extent to which extreme weather events influence ecological processes is far from understood (Jentsch et al. 2007). However, our experiment indicates that the predicted increase in rain‐on‐snow in the Arctic (Rennert et al. 2009; Hansen et al. 2014; IPCC, 2014) could have a negative impact on recruitment and shoot survival of a widespread and common Arctic tundra species. As this appears to alter within‐plant patterns of vegetative growth and likely also biomass production, a change in the frequency or magnitude of icy winters could influence vegetation community composition, trophic interactions, and ecosystem dynamics.

## Conflict of Interest

None declared.
